# Introducing Diinamic, a flexible and robust method for clustering analysis in single-molecule localization microscopy

**DOI:** 10.1017/S2633903X23000156

**Published:** 2023-07-10

**Authors:** Anne-Lise Paupiah, Xavier Marques, Zaha Merlaud, Marion Russeau, Sabine Levi, Marianne Renner

**Affiliations:** 1 Inserm UMR-S 1270, Paris, France; 2 Sorbonne Université, Paris, France; 3 Institut du Fer à Moulin, INSERM-Sorbonne Université, Paris, France; 4 Museum National d’Histoire Naturelle, CNRS UMR 7196-INSERM U1154, Paris, France

**Keywords:** clustering analysis, DBSCAN, nanodomains, PALM, STORM, super-resolution microscopy

## Abstract

Super-resolution microscopy allowed major improvements in our capacity to describe and explain biological organization at the nanoscale. Single-molecule localization microscopy (SMLM) uses the positions of molecules to create super-resolved images, but it can also provide new insights into the organization of molecules through appropriate pointillistic analyses that fully exploit the sparse nature of SMLM data. However, the main drawback of SMLM is the lack of analytical tools easily applicable to the diverse types of data that can arise from biological samples. Typically, a cloud of detections may be a cluster of molecules or not depending on the local density of detections, but also on the size of molecules themselves, the labeling technique, the photo-physics of the fluorophore, and the imaging conditions. We aimed to set an easy-to-use clustering analysis protocol adaptable to different types of data. Here, we introduce Diinamic, which combines different density-based analyses and optional thresholding to facilitate the detection of clusters. On simulated or real SMLM data, Diinamic correctly identified clusters of different sizes and densities, being performant even in noisy datasets with multiple detections per fluorophore. It also detected subdomains (“nanodomains”) in clusters with non-homogeneous distribution of detections.

## Impact Statement

Single molecule localization microscopy not only provide images with higher resolution than classical fluorescence microscopy, but the pointillistic character of its data opened a new field of biological image analysis. The possibility to “see” molecules one by one offers the perfect way to analyse the distribution of molecules, and several analytical tools were proposed to describe the formation of aggregates or clusters. However, clusters in biological samples can be very variable in size and density and the available analytical tools are, in general, effective only for a certain type of distribution. Moreover, the characteristics of clusters depend not only on the molecules themselves, but also on the labelling technique, the photo-physics of the fluorophore and the imaging conditions. We developed Diinamic, which combines different density-based analyses and optional thresholding to facilitate the detection of clusters in biological samples. By combining progressive analysis steps, Diinamic can be easily adapted to a large variety of molecular distributions. In addition, it provides the possibility to introduce biology-based criteria to describe the clustering behaviour of molecules. To help with its application, we provide cues about the strategy to follow depending on the characteristics of the dataset.

## 1. Introduction

An indisputable breakthrough in microscopy, the advent of super-resolution microscopy is also a major improvement in our capacity to describe and explain biological organization at the nanoscale. A particularly relevant question in cellular biology is to know whether molecules are randomly distributed or if they form complexes or aggregates (clusters) which may be intimately related to their function. Super-resolution imaging is being employed to analyze molecular complexes of tens to hundreds of nanometers and prompted this kind of study in living cells^(^^1^^,^^2^^)^.

Several methods have been proposed (reviewed in Khater *et al.*^(^^3^^)^). Among them, single-molecule localization microscopy (SMLM) is based on the observation of signals produced by individual fluorophores. This approach exploits the intrinsic properties of some fluorophores that allow controlling their stochastic ‘on/off’ switching or blinking. The most common SMLM methods are photoactivated localization microscopy (PALM)^(^^4^^)^, stochastic optical reconstruction microscopy (STORM)^(^^5^^–^^7^^)^, and point accumulation for imaging in nanoscale topology (PAINT)^(^^8^^)^. These approaches enable the reconstruction of an image by pointillism, with a resolution that depends basically on the signal-to-noise ratio^(^^2^^)^. Typically, the achieved resolution is of tens of nanometers, that is, a ~ 10-fold improvement with respect to the spatial resolution imposed by the diffraction limit in a conventional microscope. A visual reconstruction (called rendered image^(^^9^^)^) can be generated by convolving a Gaussian distribution of intensity (whose size is the localization precision) to each localization coordinate obtained.

SMLM offers excellent means to analyze the distribution of molecules in cells, which have been applied to many different biological systems^(^^1^^,^^2^^)^. In contrast to the images obtained with optical microscopy, SMLM techniques generate datasets of molecular coordinates in 2D or 3D. Several approaches have been proposed to analyze the spatial organization and morphology of molecular clusters from this kind of data.

Clustering analyses typically employ two different strategies, based on spatial statistics or based on density analysis. Concerning the methods based on spatial statistics, the most popular ones are the adaptation to SMLM of pair-correlation analysis^(^^10^^)^ and Ripley’s L-function^(^^11^^,^^12^^)^. As they do not provide information about the number, spatial position, and morphology of clusters, they serve to detect the presence of clustered distributions but not to describe them from a mechanistic point of view. An improvement is the analysis of topological prominence^(^^13^^)^ that informs about the shape of the cloud of points. However, these statistical approaches perform well only in the case of small clusters of relatively homogeneous size.

Density-based data mining algorithms perform better in detecting clusters in variable biological data due to their capacity to describe the spatial characteristics of individual clusters. The most extensively used is DBSCAN^(^^14^^)^, which calculates the neighboring density around each detection by counting the number of surrounding detections in a given radius. Those that are mutually reachable are considered as belonging to a cluster, and those that do not belong to clusters are considered background. Several software packages adapted this algorithm to SMLM data such as LAMA^(^^15^^)^, Clus-DoC^(^^16^^)^, and FOCAL^(^^17^^)^ (a grid-based version of DBSCAN). Unfortunately, DBSCAN performs weakly when the detections’ density in the clusters is variable among clusters, a limitation that restricts its use to datasets with homogeneous clustering. The alternatives that have been proposed introduce mesh representations to perform the density analysis. The most common strategy to create the mesh is Voronoi tessellation (SR-Tesseler^(^^18^^)^; ClusterVisu^(^^19^^)^), in which each point is on the face of a Voronoi polygon and edges are the equidistant bisectors between points. In an SMLM data set, regions with a high density of points have small polygons, so dense structures can be segmented by setting a threshold for the area of these polygons. Another improvement that was recently proposed is the application of persistence-based clustering analysis, which improves the individualization of clusters when they are close to each other^(^^20^^)^.

This variety of analytical approaches can be overwhelming for users that usually struggle to find an algorithm well adapted to their data. Moreover, SMLM techniques are affected by several technical issues that may induce various artifacts in clustering analysis; namely background noise, multiple detections of one single fluorophore over time, stage drift, or dependency on the quality of the manipulation used to prepare the sample. Some analysis strategies may be more appropriate than others depending on how the experiment was conducted and the characteristics of the targeted molecule. Our aim was to set an easy-to-use analytical protocol able to detect clusters of different characteristics, in noisy datasets with multiple detections per fluorophore. Hence, we have developed Diinamic (*Density and Image INtensity based Analysis Method for Identification of Clusters*), a modular sequence of cluster analysis that combines different density-based analyses to simplify the adaptation to different datasets. We analyzed its performance on simulated datasets and real PALM and STORM data to provide cues of which strategy to use depending on the characteristics of the dataset.

## 2. Materials and Methods

### 2.1. Simulations of SMLM data

Monte-Carlo simulations of SMLM data consisted of generating *x* and *y* coordinates for a given number of detections and were produced by a program written in MATLAB (MathWorks). Non-overlapping clusters were created by selecting the position of the first detection randomly within the simulation area (100 μm^2^). Subsequently, the other detections belonging to the cluster were positioned arbitrarily into a circular or ovoid area of the desired size. Non-clustered detections (“noise”) were introduced at random positions in the simulation area. Multiple detections of the same molecule were simulated by adding 1–50 detections around the position of the molecule. The position of these extra detections was randomly chosen following a Gaussian distribution with a mean zero and variance similar to the localization precision of our setup (20–50 nm).

### 2.2. Neuronal culture and transfection

Primary cultures of hippocampal neurons were prepared as described in Battaglia *et al.*^(^^21^^)^. Procedures were carried out in compliance with the rules of French and European regulations for the care and protection of laboratory animals (EC Directive 2010/63, French Law 2013–118, 6 February 2013). In brief, hippocampal neurons were isolated from embryonic days 18 to 20 from Sprague Dawley rat embryos of both sexes. Following attachment, cells were incubated in the described culture medium^(^^21^^)^ for up to 3 weeks at 37 °C in a 5% CO_2_ humidified incubator. Each week, one-third of the culture medium volume was renewed.

Transfections were carried out at 10 days in vitro (DIV) using TransFectin Lipid Reagent (Bio-Rad), according to the manufacturer’s instructions. The constructs Kv2.1WT-Dendra2 or Kv2.1WT-GFP derived from rKv2.1WT-GFPHis, a kind gift of J. R. Martens (University of Florida, USA). Experiments were conducted 12 days following transfection (22 DIV).

### 2.3. Immunocytochemistry for STORM

For detection of Kv2.1WT-GFP, live neurons were incubated 10 min at 37 °C with alpaca nanobodies against GFP coupled to Alexa Fluor 647 (GFP-Booster, 1/200; Proteintech) diluted in the culture medium, followed by two washes in PBS 1X. Neurons were then fixed for 10 min at −20 °C in 100% methanol and washed in PBS 1X.

For detection of Kv2.1WT-Dendra2, neurons were fixed for 15 min at room temperature (RT) in paraformaldehyde (4% w/v; Thermo Scientific Chemicals) enriched in sucrose (4% w/v; Sigma-Aldrich) and washed in PBS 1X. Neurons were then incubated for 1 hr at RT with rabbit primary antibodies against Dendra2 (1/200; Antibodies Online). After 1 hr incubation at RT in PBS 1X supplemented with goat serum (PBS-GS; 10% v/v; Invitrogen), neurons were incubated for 30 min at RT with purified donkey anti-rabbit antibodies coupled to Alexa Fluor 647 (1/250; Jackson Immunoresearch). For both incubations, antibodies were diluted in PBS-GS 10%.

For detection of GABA_A_ receptors, live neurons were incubated for 20 min at 4 °C with rabbit primary antibodies against the α1 subunit (1/500; Synaptic System) diluted in MEM-R (Minimum Essential Medium supplemented with HEPES 20 mM, Na^+^ pyruvate 1 mM, glucose 20% w/v, glutamine 2 mM and B27 1X; Invitrogen), followed by several washes in MEM-R. Subsequently, neurons were fixed for 10 min at RT in paraformaldehyde enriched in sucrose and washed in PBS 1X. Neurons were then incubated for 45 min at RT with secondary purified donkey anti-rabbit antibodies coupled to Alexa Fluor 647 (1/300; Jackson Immunoresearch) diluted in PBS-GS (3% v/v; Invitrogen) and washed in PBS 1X.

Coverslips were kept in PBS 1X until imaging.

### 2.4. Super-resolution imaging

Super-resolution imaging on fixed samples was conducted on an inverted N-STORM Nikon Eclipse Ti microscope with a 100X oil-immersion objective (NA 1.49) and an Andor iXon Ultra 897 EMCCD camera (image pixel size, 105 nm), using specific lasers for PALM imaging of Dendra2 (405 and 561 nm) and STORM imaging of Alexa 647 (405 and 640 nm). In the case of PALM and STORM imaging on the same sample, first was taken the acquisition of Alexa 647 images without reactivation of the fluorophores with the 405 nm laser and then was acquired the Dendra2 images as usual. For STORM, samples were imaged in a PBS-based oxygen-scavenging buffer containing imaging buffer (Tris 100 mM, NaCl 20 mM, pH 8), glucose 40% (w/v; Sigma-Aldrich), PBS 1X, cysteamine hydrochloride (MEA; Sigma-Aldrich), catalase 5 mg/mL (Sigma-Aldrich) and pyranose oxidase 200 U/mL (Sigma-Aldrich). Catalase was diluted in MgCl 4 mM, 2 mM EGTA, and PIPES 24 mM (Sigma-Aldrich, pH 6.8). Pyranose oxidase was diluted in the same buffer supplemented with glycerol (50% v/v). Videos of 30,000 frames were acquired at a frame rate of 20 msec. The *z* position was retained during the acquisition by a Nikon Perfect Focus System.

### 2.5. Single-molecule localization

Single-molecule localization and 2D image reconstruction was performed as described in Specht *et al.* ^(^^22^^)^ by fitting the PSF of spatially separated fluorophores to a 2D Gaussian distribution. Poorly localized peaks (fitting R^2^ < 0.7) were not included for further analysis. To correct multiple detections coming from the same Dendra2 molecule, we identified detections occurring in the vicinity of space (2 σ) and time (15 s) as belonging to the same molecule^(^^21^^)^. The drift of the stage was corrected using 100 nm multicolor fluorescent beads (TetraSpeck, 1/300; Invitrogen) to follow the movement through the frames; and was nullified by superimposing the molecular localizations of each frame. Rendered images were obtained by superimposing the coordinates of single-molecule detections, which were represented with 2D Gaussian curves of unitary intensity and SDs representing the localization precision^(^^9^^)^.

### 2.6. Clustering analysis

All data analyses were performed using custom routines in MATLAB (MathWorks) and executed typically in a PC equipped with an IntelCore i7 processor (1.69 GHz) and 16 Gb of RAM. The DBSCAN program was sourced from Pageon *et al.* ^(^^16^^)^ and adapted to be run on MATLAB. The calculation of the adjusted rand index (ARI) and intersection over union (IoU) scores was implemented in MATLAB following the algorithms described in Nieves *et al.*^(^^23^^)^. Clustering detection was carried out on regions of interest (ROIs) drawn on top of pointillistic images constructed from the coordinates of detections. A package with all the tools (ROI selection and clustering analysis) for executing Diinamic-R and Diinamic-V is freely available at https://github.com/mlrennerfr/Diinamic. This package includes Graphic User Interfaces (GUIs), a user manual, and a tool helping the optimization of parameters.

#### 2.6.1. DBSCAN

Starting at a random detection of the dataset, this detection is considered as a “core” point of a cluster if a minimum number of points were found around it, within the distance of the search radius (*epsilon*). The detections within the search radius then belonged to the cluster. By iteration, the algorithm proceeds similarly for all detections of the dataset. Ultimately, detections that do not belong to any cluster are considered as non-clustered detections.

#### 2.6.2. Diinamic-R and Diinamic-V

Detections were initially sorted into belonging to candidate clusters or not depending on their localization with respect to a segmentation mask. This mask was created by either thresholding the pixel intensity of the corresponding rendered image (Diinamic-R) or by evaluating the size of polygons resulting from a Voronoi tessellation (Diinamic-V) calculated using the “voronoi” function of MATLAB. The clusters’ borders were defined by the “boundary” function of MATLAB.

Candidate clusters were retained if: (a) their density, calculated in a pixel-wise manner, exceeded a threshold; (b) their sizes were between given thresholds (minimum and maximum). Thresholds were chosen considering the expected number of detections per molecule, the size of the molecule and the expected size of clusters given other microscopy data already published.

### 2.7. Statistical analysis

All statistical analyses were performed using GraphPad Prism 9 (Dotmatics). Statistical significance was determined using Kruskal–Wallis tests followed by Dunn’s multiple comparison tests. Differences were considered not significant for *p-values* > .05, while significant values were indicated as follows: *: *p* < .05, **: *p* < .01, ***: *p* < .001 and ****: *p* < .0001.

## 3. Results

**Figure 1.**
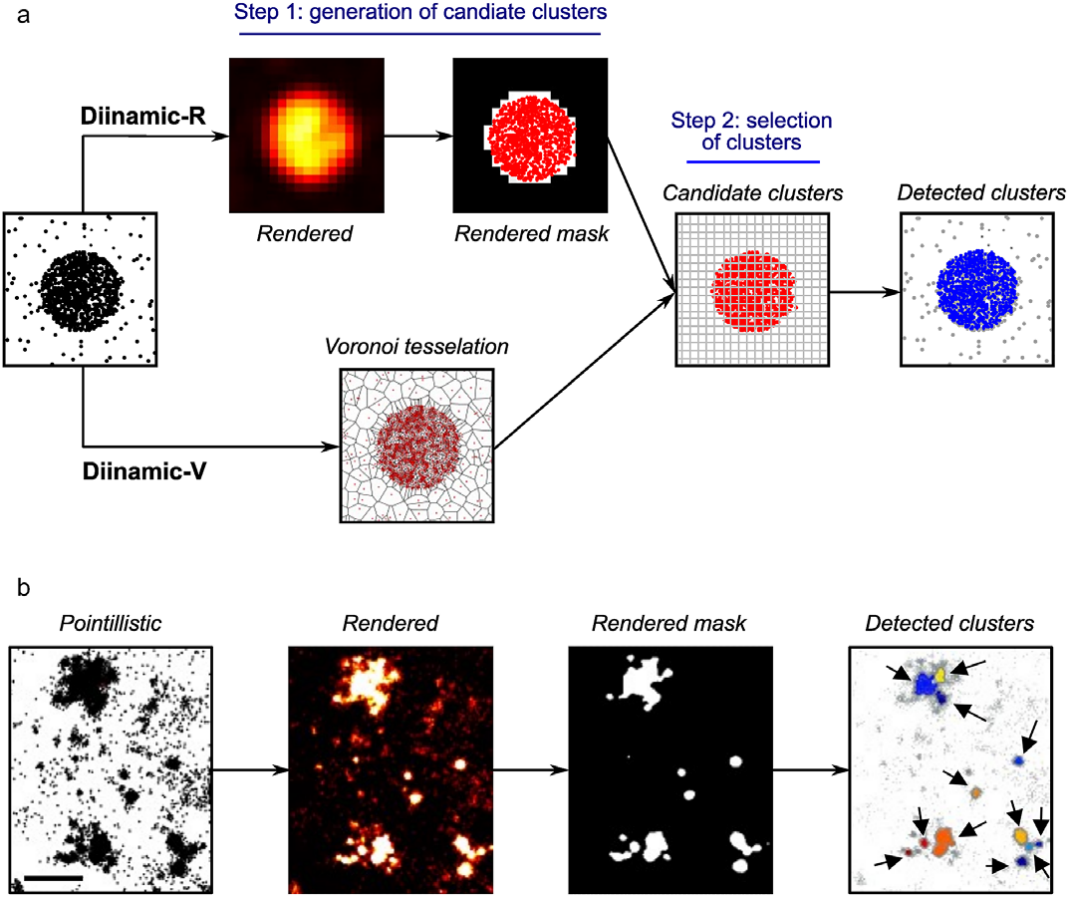
Protocol for clustering analysis on SMLM data using Diinamic-R or Diinamic-V. (a) Schematic representation of the analysis phases on a simulated cluster. Diinamic-R relies on the segmented rendered image to select candidate clusters whereas Diinamic-V uses a Voronoi tessellation. Pixels that bear enough density of detections (Diinamic-R) or Voronoi tesserae (polygons) that are small enough (Diinamic-V) are used to create candidate clusters by coalescence. Candidate clusters are analyzed on a grid created from the pixel size of rendered images, and they are retained if they fulfill density and size criteria. (b) Application of the Diinamic-R protocol on PALM data of Dendra2-tagged Kv2.1 channels in hippocampal neuron cultures. The pointillistic image (left) represents the coordinates of detections, which are used to create a rendered image of detections (“Rendered”, see Materials and methods). After segmenting the rendered image with an intensity threshold, the rendered mask is used to preselect pixels and candidate clusters. The final result (right panel, “Detected clusters”) shows each retained cluster in a different color (arrows). Note the variable size of clusters that can be adjacent to other clusters. Scale bar: 300 nm.

We propose a new cluster identification algorithm, which performs a modular density-based analysis. The analysis was performed in two (cluster detection) or three phases (cluster and intra-cluster subdomain detection). The first phase preselected pixels or detections that were likely to belong to clusters, using density-based criteria (Figure 1a). They were then used to create candidate clusters by coalescence. Phase two retained the candidate clusters that fulfilled user-defined parameters (minimum density, and minimum and maximum size). The density threshold was calculated considering the size of the molecule of interest and the labeling method (number of expected fluorophores per molecule). The third phase, optional, sought subdomains with different densities in the selected clusters.

The purpose of the first phase was to eliminate low-density and low-intensity detections, reducing spurious cluster detection and computational burden by eliminating irrelevant detections. These detections were considered as noise originating from non-clustered molecules, from autofluorescence or out-of-focus fluorophores.

There were two options for the first phase: Diinamic-R or Diinamic-V (Figure 1a). The Diinamic-R algorithm selected candidate pixels by intensity-based segmentation of the rendered image. The pixel size of this image determined the grid used to calculate density. In addition to the intensity threshold, we applied a density threshold to each pixel if needed. These two thresholds were chosen to eliminate pixels with a value of intensity and/or a number of detections too low to be considered part of a cluster. Those retained were used to create candidate clusters (Figure 1b). Alternatively, Diinamic-V selected candidate clusters by Voronoi tessellation, using the area of the generated polygons for the selection (Figure 1a). Voronoi polygons below a size threshold were retained to create candidate clusters.

The second phase verified whether the candidate clusters fulfilled the criteria regarding their minimum size and internal density (i.e., to remove false clusters due to multiple detections of one single molecule) or maximum size (i.e., to remove coalescent clusters that could not be correctly defined). Density was calculated in a pixel-wise manner, using the pixel size of the rendered image (Figure 1a).

### 3.1. Performance analysis on point classification and geometric overlap

We first compared the performance of Diinamic-R and Diinamic-V on various sets of simulations of SMLM data. In a recent article, Nieves *et al.*^(^^23^^)^ proposed a framework to evaluate SMLM cluster analysis algorithms, with simulated scenarios representing diverse situations of density of detections and size of clusters in a squared area of 2 μm × 2 μm. They also proposed two metrics, ARI, which analyzes the classification of detections into the same clusters as the ground truth; and IoU, which analyzes the overlap between the cluster areas in the output and the ground truth (here, the correct result). Perfect match provides values equal to one. We therefore analyzed their scenarios with Diinamic-R and Diinamic-V.

Scenarios comprised different combinations of clustered and randomly distributed detections ^(^^23^^)^. They included simulations of sparse data with very small clusters (Scenario 4); clusters of regular size, well separated, with different densities of homogeneously distributed detections inside and outside clusters (Scenarios 2, 3, and 8) or clusters surrounded by non-homogeneous distribution of non-clustered detections (Scenario 10); non-round clusters (Scenario 6) and clusters of different sizes (Scenarios 7, 8, and 9). Conditions were particularly difficult for clustering analysis in Scenarios 5, 6, 7, and 9, where clusters could overlap, and sometimes non-clustered detections appeared inside the perimeter of the cluster (Scenario 7). The nine scenarios were simulated in the absence (ground truth scenarios) or presence (multiple blinking scenarios) of multiple detections per each original molecule simulated. In the latter case, each single molecule was replaced by a cloud of ~4–5 detections on average ^(^^23^^)^.

As the real clusters were known, we optimized parameters to obtain results the closest possible to them. The execution times for the ground truth scenario with the lowest number of detection (400 detections, Scenario 4) was 0.26 s for Diinamic-R and 0.98 s for Diinamic-V, whereas for the one with the largest number of molecules (3000 detections, Scenario 9), the analysis took 0.35 s for Diinamic-R and 6.02 s for Diinamic-V.

Both Diinamic-R and Diinamic-V performed well in most scenarios (Tables 1 and 2, Supplementary Figures S1 and S2). Not surprisingly, the worse ARI scores were obtained in Scenarios 5, 6, 7, and 9, where there is an important overlap of clusters; then the attribution of detections to the correct cluster was less performant. In the case of IoU scores, the worst was Scenario 4. This indicated, as already reported^(^^23^^)^, that the mapping of the borders of the cluster was difficult in sparse data where clusters were composed of few detections (5 in this case). Multiple blinking increased ARI and reduced IoU scores, as expected (Tables 1 and 2, Supplementary Figures S1 and S2).

**Table 1.** Performance analysis of Diinamic-R and Diinamic-V against Ground truth scenarios 2–10 from Nieves *et al.*^(^^23^^)^.

	ARI (mean ± SD)		IoU (mean ± SD)	
Ground truth scenario #	Diinamic-R	Diinamic-V	Diinamic-R	Diinamic-V
2	0.88 ± 0.06	0.82 ± 0.10	0.87 ± 0.02	0.72 ± 0.02
3	0.93 ± 0.05	0.93 ± 0.05	0.84 ± 0.04	0.69 ± 0.02
4	0.87 ± 0.08	0.70 ± 0.12	0.58 ± 0.08	0.46 ± 0.06
5	0.51 ± 0.06	0.53 ± 0.06	0.77 ± 0.03	0.67 ± 0.01
6	0.74 ± 0.10	0.65 ± 0.10	0.83 ± 0.03	0.81 ± 0.02
7	0.68 ± 0.10	0.50 ± 0.13	0.71 ± 0.03	0.68 ± 0.03
8	0.87 ± 0.89	0.81 ± 0.10	0.79 ± 0.04	0.64 ± 0.04
9	0.58 ± 0.13	0.59 ± 0.14	0.80 ± 0.02	0.78 ± 0.02
10	0.89 ± 0.06	0.83 ± 0.09	0.82 ± 0.05	0.67 ± 0.03

*Note.* Results were scored using ARI and IoU scores using the same optimized parameters for all the simulations (*n* = 50) from each scenario.

**Table 2.** Performance analysis of Diinamic-R and Diinamic-V against scenarios 2–10 from Nieves *et al.*^(^^23^^)^, simulating multiple blinking of fluorophores.

	ARI (mean ± SD)		IoU (mean ± SD)	
Multiple blinking scenario #	Diinamic-R	Diinamic-V	Diinamic-R	Diinamic-V
2	0.91 ± 0.07	0.91 ± 0.08	0.75 ± 0.06	0.57 ± 0.09
3	0.92 ± 0.07	0.94 ± 0.06	0.64 ± 0.08	0.61 ± 0.05
4	0.91 ± 0.08	0.91 ± 0.07	0.39 ± 0.07	0.36 ± 0.06
5	0.65 ± 0.07	0.61 ± 0.06	0.70 ± 0.04	0.79 ± 0.02
6	0.71 ± 0.10	0.73 ± 0.11	0.85 ± 0.03	0.82 ± 0.02
7	0.74 ± 0.12	0.67 ± 0.13	0.65 ± 0.07	0.66 ± 0.04
8	0.92 ± 0.07	0.88 ± 0.09	0.65 ± 0.05	0.66 ± 0.04
9	0.60 ± 0.15	0.63 ± 0.15	0.84 ± 0.03	0.79 ± 0.03
10	0.91 ± 0.06	0.92 ± 0.06	0.78 ± 0.06	0.68 ± 0.04

*Note.* Results were scored using ARI and IoU scores using the same optimized parameters for all the simulations (*n* = 50) from each scenario.

To compare the versatility of Diinamic algorithms with respect to those already tested on the same scenarios (DBSCAN, ToMATo, KDE, FOCAL, ClusterViSu, CAML, and SR-Tesseler)^(^^23^^)^, we considered the number of scenarios in which each algorithm obtained a score of at least ~0.8 (good), between ~0.6 and ~ 0.8 (acceptable) or below ~0.6 (bad) (Supplementary Table S1). As distributions of ARI and IoU scores could be large, we chose the category in which most of the distribution of the score was situated, and we counted the number of scenarios with acceptable or good scores (Table 3)^(^^23^^)^. Diinamic algorithms were overall the most versatile, as they obtained acceptable or good results in more scenarios than the other algorithms.

**Table 3.** Versatility of Diinamic-R and Diinamic-V, evaluated by counting the number of scenarios with acceptable or good scores (scores above ~0.6).

	Number of scenarios with scores > ~0.6				
	ARI		IoU		Sum
Algorithm	Ground truth scenarios	Multiple blinking scenarios	Ground truth scenarios	Multiple blinking scenarios	
Diinamic-R	7	8	8	8	31
Diinamic-V	7	8	8	8	31
DBSCAN	7	5	6	7	25
ToMATo	6	5	6	5	22
KDE	5	4	4	3	17
CAML	5	5	6	0	16
FOCAL	4	1	4	1	10
SRTesseler	4	3	1	2	10
ClusterVISU	4	4	0	0	8

*Note.* Column “Sum” is the sum for both scores in Ground truth and Multiple blinking scenarios.

To extend testing to situations with high density of detections and larger clusters, as the ones that we observe in some of our experiments, we created additional simulated scenarios.

### 3.2. Diinamic-R and Diinamic-V better defined the contour of clusters even under high noise conditions

**Figure 2.**
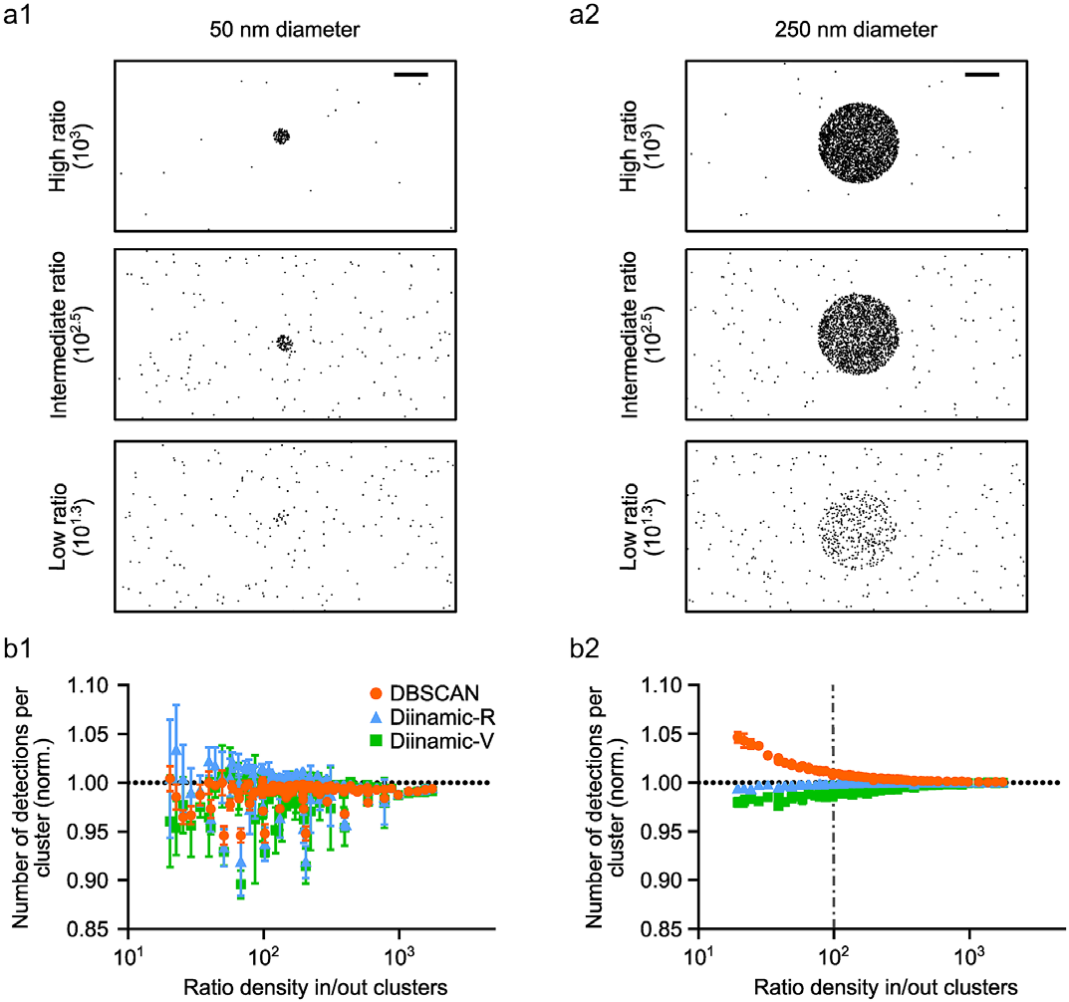
Performance of DBSCAN, Diinamic-R, and Diinamic-V in finding the borders of clusters depending on the density of background noise. (a) Examples of a simulated cluster of small (50 nm; a1) or large (250 nm; a2) diameter, surrounded by non-clustered detections (“noise”). The simulations contained 10 clusters with the same characteristics. The density of detections in and out of clusters was variable to vary the ratio cluster density/noise density. Scale bar: 100 nm. (b) Quantifications of the number of detections per detected cluster for small (b1) and large-sized (b2) simulated clusters with respect to the ratio between the density in and out of clusters (Ratio density in/out clusters) for DBSCAN (orange circles), Diinamic-R (blue triangles), and Diinamic-V (green squares). The horizontal discontinuous line represents the ground truth. Mean ± SD, *n* = 3 independent simulations.

We tested systematically the capacity of Diinamic algorithms to define the borders of clusters if they were surrounded by randomly distributed detections (noise) at different densities. We thus simulated different combinations of detection densities in and out clusters. The density of detections within clusters was homogeneous. (Figure 2a).

A first series of simulations was created containing 10 clusters of identical shape with a diameter of 50 nm (Figure 2a1) or 250 nm (Figure 2a2). In each simulation, all clusters had a given density, ranging from 8000 to 70000 detections/μm^2^. The density of random detections ranged from 0 to 40000 detections/μm^2^ (Figure 2a1,a2). All the parameters of density and size used as thresholds for clustering analysis corresponded to the ground truth (here, the correct result).

For comparison purposes, we calculated the ratio of detection densities in and out of clusters for each set of simulations and we assessed the quality of cluster detection in relation to this ratio by counting the number of detections assigned to each cluster.

Given the results obtained above, we compared the results of Diinamic-R and Diinamic-V to those of DBSCAN, which showed a good level of adaptability to different scenarios (Table 3). DBSCAN and both Diinamic algorithms were able to detect the correct number of clusters across all ratios (not shown). No cluster was detected among the randomly distributed detections, even at high density (not shown).

As the number of detections changed from one simulation to the other, we divided the number of detections in each cluster by the real value to obtain a normalized number of detections (the ground truth is then equal to 1). Figures 2b1 and b2 show the normalized number of detections for small and large clusters, respectively. As expected, all methods performed correctly when defining the borders of the clusters in case of low noise (high ratio), thus they correctly determined the number of detections belonging to the cluster in these conditions (ratio > 10^3^; Figure 2b1 and b2). In the presence of noise, however, their performance was affected by the size of clusters, providing results that roughly underestimated the number of detections by up to ~10% in the case of small clusters (Figure 2b1).

The result provided by Diinamic-R was overall closer to the ground truth for all ratios, in particular for the larger clusters (Figure 2b2). DBSCAN performed well for small clusters but over-evaluated the number of detections in the case of large clusters and low ratios (~^5^%). As a rule of thumb, we found that DBSCAN did not perform as well as Diinamic when the difference in density inside and outside clusters was less than 10^2^.

### 3.3. Diinamic-V was less affected by multiple detections of the same molecule

An important drawback in SMLM is the presence of multiple detections of the same fluorophore. Each fluorophore may be randomly detected several times (i.e., in multiple images of the acquisition, consecutive or not), generating a cloud of detections that could be easily considered as a cluster of molecules. This artifact is maximal when the labeling strategy results in a high number of fluorophores attached to the same molecule. We considered as the worst situation the STORM imaging of a large molecule containing several subunits, that is, potentially having several primary and secondary antibodies bound to it, together with the use of a secondary antibody bearing several fluorophores.

**Figure 3.**
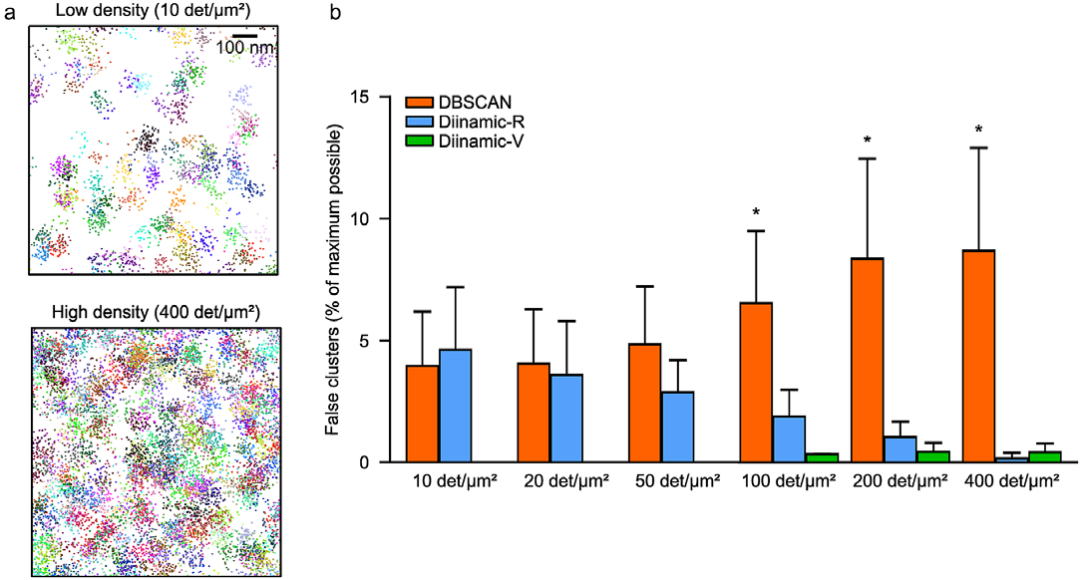
Performance of DBSCAN, Diinamic-R, and Diinamic-V in avoiding the detection of false clusters arising from multiple detections of randomly distributed molecules. (a) The positions of randomly distributed molecules at low (top panel) or high (bottom panel) density were overlaid by a cloud of detections to simulate multiple detections of each molecule. Each group (initial detection plus the cloud of multiple detections) is depicted in a different color. Scale bar: 100 nm. (b) Proportion of false clusters detected (as % of the maximum possible, which is the number of molecules) by the three analysis methods, for simulations with the indicated density of molecules. Mean ± SD, *n* = 3 independent simulations, KW test, and Dunn post-hoc with respect to the ground truth, * *p* < .05.

To assess the robustness of cluster detection in these conditions regarding the parameters used in the analysis, we simulated randomly distributed detections, and we added a cloud of extra detections around the original detection (Figure 3a). The position of these extra detections was randomly chosen following a Gaussian distribution with a mean zero and a variance equivalent to a typical localization precision (20–50 nm). We varied the number of extra detections (0 to 50) to reach densities that can be observable in real experiments. We empirically optimized each user-given parameter for the three analyses to sift out as many false clusters as possible. Results are expressed as % of the maximum number of possible false clusters (the number of simulated molecules).

In sets with only randomly distributed detections, none of the algorithms detected any cluster (not shown). However, as soon as simulations included multiple detections, DBSCAN performed poorly, always detecting 5–10% of false clusters (Figure 3b). Diinamic-R displays a tendency to collect some false clusters at low densities of detections (non-significant) but performed better than DBSCAN for higher densities (Figure 3a,b). Diinamic-V always showed more reliable results being able to reject all false clusters in the range of 0 to 50 detections/μm^2^ (Figure 3a,b).

Therefore Diinamic-V was overall the best choice to overcome false cluster detection, although Diinamic-R performed notably well at high density of detections.

## 4. Diinamic-R and Diinamic-V Performed Better than DBSCAN on Simulations of Mixed Populations of Clusters

**Figure 4.**
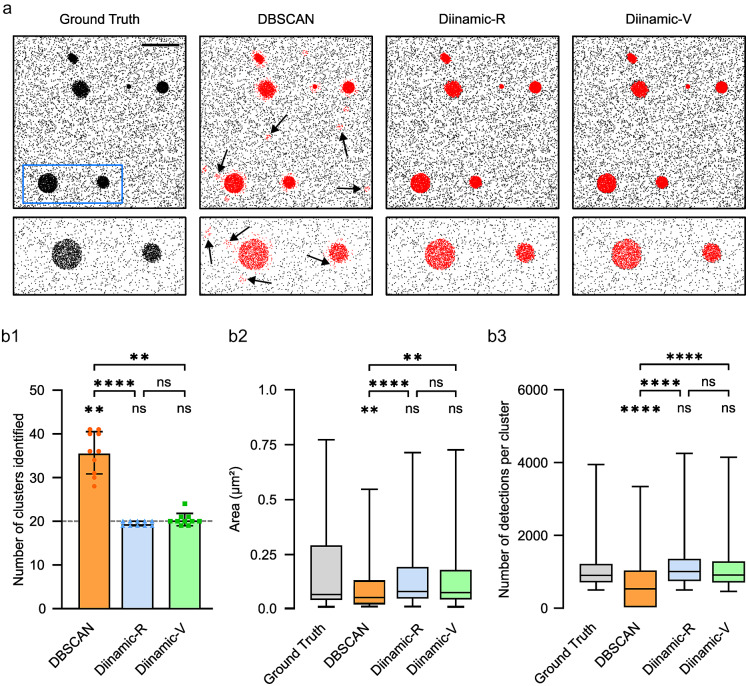
Performance of DBSCAN, Diinamic-R, and Diinamic-V in detecting clusters in a mixed population of clusters of different sizes and densities, in the presence of multiple detections. Simulated data contained 20 clusters of different sizes and densities, surrounded by randomly distributed detections. (a) Pointillistic images of an example of simulation and the clusters detected by the three analyses (in red). Blue rectangle: area shown with higher magnification in the lower panels. Note the detection of small and false clusters by DBSCAN (black arrows). Scale bar: 1 μm. (b) Quantifications of the detected clusters. (b1) Number of clusters (the horizontal line shows the ground truth). Mean ± SD. (b2) Area. Median and 5%–95% IQR. (b3) Number of detections. Median and 5%–95% IQR. *n* = 10 independent simulations. KW test and Dunn post-hoc, ns, not significant; **, *p* < .01; ****, *p* < .0001.

Data obtained on biological samples show clusters of molecules that often have non-homogeneous spatial characteristics and densities, which complicates the choice of thresholds for their detection. We therefore simulated images containing clusters of different sizes and densities. Simulations contained 20 round or ovoid clusters of sizes ranging from 100 to 1,000 nm in diameter, with densities of 4,000 to 60,000 detections/μm^2^. A moderate quantity of noise (110 detections/μm^2^) was added to reproduce a typical background noise. In the same line, 0 to 10 extra detections were added to each detection to simulate multiple detections (Figure 4a). We used empiric parameters for each analysis, chosen to obtain results that were the closest to the ground truth.

As before, we evaluated the number of clusters that were detected, their number of detections, and their area. In the case of DBSCAN, it was impossible to find a set of parameters that correctly picked up all the true clusters: either the analysis missed small and low-density clusters, or it detected false clusters arising from multiple detections. It often included detections out of high-density clusters (Figure 4a, inset). Figure 4b shows the results obtained with the parameters needed to detect at least all the real clusters. DBSCAN systematically identified more clusters than what was expected (Figure 4b1). Diinamic-R and Diinamic-V were both able to detect the right number of clusters (Figure 4b1). Regarding the area of clusters and the number of detections per cluster, DBSCAN results were again significantly different from the ground truth, whereas Diinamic-R and Diinamic-V provided distributions that were not significantly different from the ground truth (Figure 4b2 and b3).

Overall, we observed that DBSCAN had the tendency to fragment large and less dense clusters or identify false clusters distribution arising from multiple detections. In that regard, Diinamic-R and Diinamic-V efficiently bypassed background noise, size, and shape differences to identify a reliable number of clusters with area and number of detections not significantly different from the ground truth.

### 4.1. E*xample of application on PALM and STORM data*

We wondered how the different labeling strategies of SMLM might affect cluster detection. PALM may under-sample structures such as clusters if not all the molecules present in the structure bear a mature fluorescent protein. On the other hand, STORM may over-sample because antibodies can carry more than one fluorophore, and there could be more than one antibody per molecule.

To compare properly the clustering detection on PALM and STORM acquisitions, we transfected neurons with a Dendra2-tagged Kv2.1 chimera (Kv2.1WT-Dendra2). Kv2.1 is a tetrameric potassium channel known to form large clusters in neurons^(^^24^^)^. Before the imaging session, we immunolabeled Dendra2 with Alexa647 fluorophores so we could perform PALM and STORM to obtain two sets of SMLM data of the same molecule on the same sample (see Materials and Methods section).

**Figure 5.**
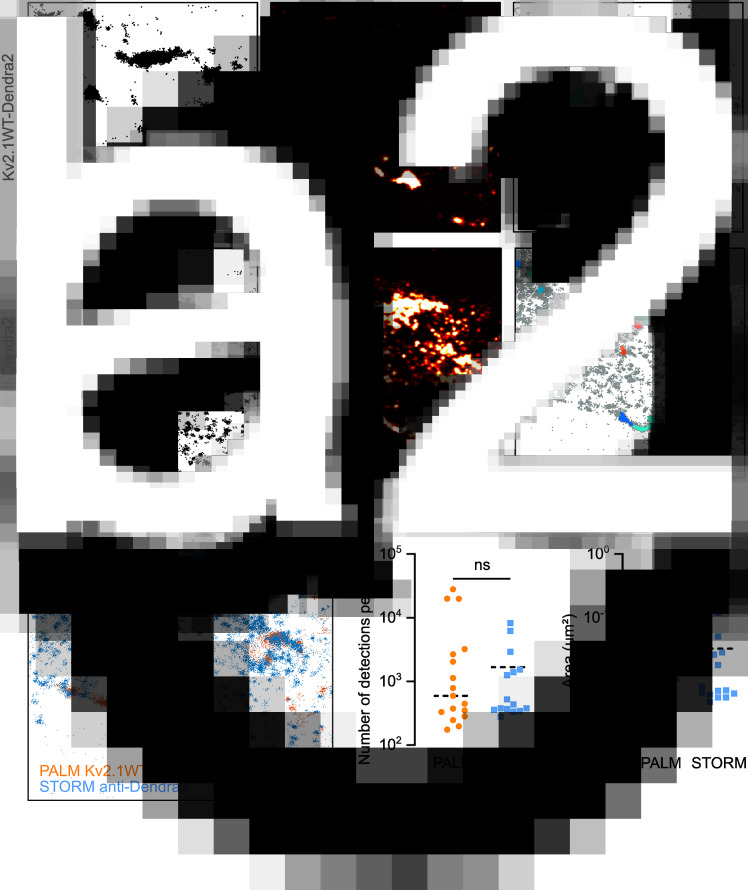
Clustering analysis in PALM vs STORM data. Pointillistic images (a1, b1); rendered images (a2, b2) and cluster detection (a3, b3) of a PALM (a) and STORM (b) dataset obtained on the same molecules (Kv2.1WT-Dendra2, labeled with Alexa 647-coupled antibodies anti-Dendra2) of the sample. In a3, b3, each detected cluster is depicted with a different color; all the other detections appear in grey. Scale bar: 2 μm. (c) Overlay of pointillistic images in a1, b1. Scale bar: 2 μm. (d) Cluster detection results (number of detections per cluster, d1; area of clusters, (d2) of the data in a3, b3.

Figure 5a–c shows an example of the data obtained. Some clusters of PALM detections overlap as expected with those of STORM detections, while some others were present only in one set of data, due to the different labeling strategies (Figure 5a–c). STORM data showed a multitude of small clusters that were not observable in PALM data. Thus, we considered them as being multiple detections of the same molecule (i.e., multiple fluorophores/antibodies on the same molecule). Some of the clusters observed with STORM seemed to be larger than in PALM data: this could be a consequence of placing the fluorophore on the secondary antibody, thus at a longer distance from the molecule of Kv2.1.

Both Diinamic-R and Diinamic-V (not shown) succeeded in identifying the same clusters in these datasets. However, as the execution time could be 20 times longer for Diinamic-V in the case of large datasets, we privileged Diinamic-R to run this test.

To set the minimum thresholds for Diinamic-R, we considered the structure of Kv2.1, the labeling method, and the expected size of clusters. As we could not know how many chimeric subunits were present per cluster (tetramers could contain both endogenous and chimeric subunits), we chose to use a low threshold for the number of detections (150). We calculated this number using the strategy described in Patrizio *et al.*^(^^25^^)^ to assess the number of detections expected for one molecule.

Given the worst localization precision in this experiment (~30 nm for Dendra2 detection), and the possible enlargement of clusters due to the use of antibodies, the minimum size of the cluster was 50 grid units (5000 nm^2^, a square of ~70 nm in side) for PALM data and 70 grid units for STORM data. Given all the above considerations, the minimum density inside clusters was set to 2 detections/grid unit.

The most important difference between the parameters for PALM and STORM data was the intensity threshold to segment the rendered image (PALM: 0.5% of the maximum intensity; STORM: 15%) and the use of a minimum density per pixel (2) in case of STORM data. These differences were due to the different detection density ratios in and out clusters (Figure 5a,b).

The number of clusters detected was different (PALM: 17; STORM: 15), in agreement with the fact that some clusters were not observable in both datasets. However, we obtained a distribution of the number of detections per cluster and cluster surfaces that were not significantly different (Figure 5d). Therefore, even if the labeling method had a strong influence on the characteristics of the dataset, it is possible to set parameters that provide concordant results for the same molecule.

### 4.2. Improving cluster detection by combining Diinamic with DBSCAN

**Figure 6.**
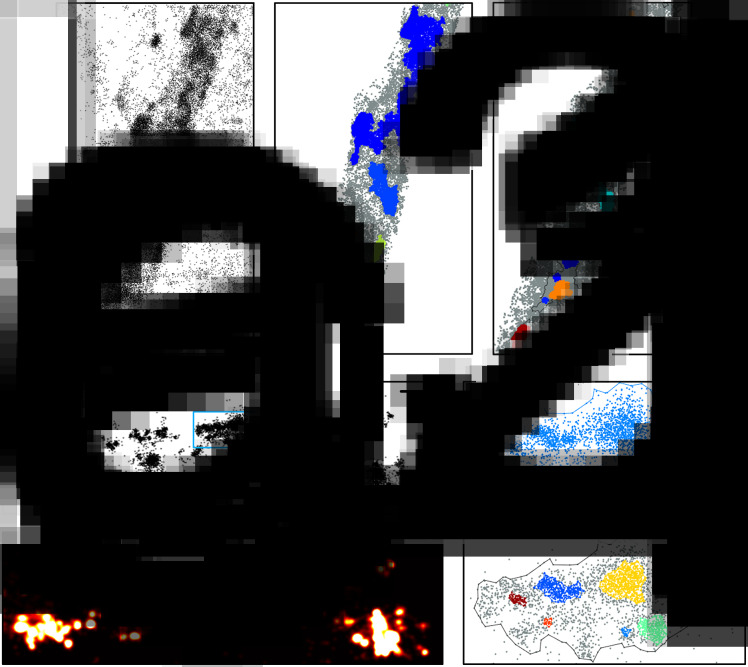
Detection of clusters and subdomains within clusters on SMLM data. (a) SMLM detections (pointillistic image, a1) and cluster detection in an example of dense PALM data for Kv2.1 in a cultured hippocampal neuron. (a2) Clusters found by Diinamic-R, depicted in different colors. (a3) Second detection of clusters by DBSCAN (subdomains), inside the clusters detected by Diinamic. Scale bar: 1 μm. (b) Pointillistic (b1) and rendered (b2, false colors) STORM images of the α1 subunit of GABA_A_R at the surface of a cultured hippocampal neuron. The blue rectangle indicates the region zoomed in C. Scale bar: 200 nm. (c) Higher magnification of a cluster detected in b1 (c1) and the subdomains found (c2). In c2, each subdomain appears in a different color. Detections in the cluster but not in subdomains appear in grey. The contour indicates the border of the cluster. Scale bar: 100 nm.

In the case of dense datasets with variable density of detections, applying the same thresholds to different regions of the cell may provide results that are not satisfactory. Figure 6a shows an example of dense labeling of Kv2.1WT-Dendra2. Clusters recognizable by eye were merged by the analysis (dark blue in Figure 6a2). To improve the analysis in these conditions, we performed two rounds of selection. In the first round, we selected clusters as described before (Figure 6a2) and in the second round, we looked inside these clusters to identify areas with higher density (subdomains). We obtained the best results using DBSCAN for the second round. To cope with variable density from one cluster to the other, we calculated the search distance *epsilon* from the mean distance between detections in the cluster. As shown in Figure 6a3, this strategy identified well the regions with higher densities that were observable by the eye in the pointillistic image (Figure 6a1).

Indeed, this double-detection strategy was also able to detect sub-synaptic domains (nanodomains) of GABA_A_ receptors in synapses. Super-resolution microscopy revealed that neurotransmitter receptors, including the GABA_A_ receptors^(^^26^^–^^28^^)^, can form sub-synaptic domains in synapses whose existence can tune the efficacy of synaptic transmission (reviewed in Yang and Specht^(^^29^^)^, Maynard *et al.*^(^^30^^)^). We analyzed a STORM dataset of the α1-subunit of GABA_A_ receptor, which displayed a non-homogeneous clustered distribution in synapses^(^^26^^)^, and a mixed clustered and non-clustered distribution outside synapses. Figures 6b1 and b2 show respectively the pointillistic and rendered images of an ROI on a dendrite of a hippocampal neuron. Figure 6c shows the detail of one of the detected clusters using Diinamic-R (Diinamic-V provided the same results, not shown) before (Figure 6c1) and after (Figure 6c2) the detection of subdomains with DBSCAN.

## 5. Discussion

Over the last decades, super-resolution microscopy has remarkably evolved, enabling the study of the organization of molecules with a resolution of a few tens of nm. SMLM revealed sub-diffractive protein structures in diverse situations, such as T-cell signaling, the molecular architecture of the nuclear pore complex, sub-synaptic domains (“nanodomains”) of receptors, actin structures in axons and the arrangement of nucleosomes on chromatin fibers ^(^^14^^,^^31^^,^^32^^)^. Besides the possibility to generate rendered images that are better resolved than regular optical fluorescent images, the pointillistic character of SMLM data opens a new field of biological image analysis. Indeed, rendered images hinder important information about the quality of labeling and the real density of detections, which may mislead comparisons between different experimental conditions^(^^14^^)^. The analysis of detection coordinates is preferable as it provides information not only about the distribution of molecules but also about the quality of the data, helping to choose the right clustering algorithm and providing a quantitative imaging tool at the nanoscale applicable to living cells.

Despite the power of SMLM, which theoretically provides the position of every single molecule, its application to biological samples is not straightforward. Typically, the difficulty to decide whether a cloud of detections is a cluster of molecules resides in the fact that the characteristics of these clusters depend not only on the molecules themselves (their size and their local concentration) but also on the labeling technique (number of fluorophores attached to the molecule, distance between the fluorophore and the molecule of interest, the proportion of molecules that bear a fluorophore), the photo-physics of the fluorophore (blinking) and the imaging conditions (laser power and acquisition protocol). In addition to this, spurious detections are coming from autofluorescence and out-of-focus signals.

### 5.1. Diinamic, a modular method for cluster analysis on SMLM data

Diinamic is a modular sequence of cluster detection steps that couples a space fragmentation method using an intensity-thresholded rendered image or Voronoi tessellation, with a local density calculation. The space fragmentation phase helped to reduce the relative importance of low-intensity signals arising from out-of-focus fluorophores or autofluorescence. Consequently, it also reduced the amount of irrelevant data. The use of a grid-based selection, with a grid size based on the pixel size of rendered images, introduces a density criterion that also considers the localization precision. The use of the smallest possible grid size also made the choice of the size unambiguous, and the analysis independent of the shape and size of clusters.

In the variety of situations that we tested here, Diinamic-R and Diinamic-V managed to detect the proper number of clusters and to provide trustable distributions of area and number of detections per cluster showing a remarkable versatility.

The strategy of space fragmentation using a grid has been already implemented by FOCAL^(^^17^^)^. However, the use that Diinamic-R and FOCAL make of this grid is different. Whereas FOCAL enhances the density difference in and out clusters by convolving the number of detections of each element of the grid (SMLM pixel) with a 3x3 sum filter, Diinamic-R does not modify the number of detections per pixel, as the enhancement is performed by the intensity threshold applied to the rendered image. Thus, FOCAL blurs the borders whereas Diinamic-R sharpens them.

Indeed, Diinamic algorithms mapped the borders better than DBSCAN even in difficult situations with clusters whose internal density was only 10 to 100 times higher than the density of detections in the surroundings. Therefore, Diinamic-R and Diinamic-V proved to be more robust to artifacts arising from the uneven distribution of non-clustered detections or the uneven distribution of background noise within a sample or between samples. They also performed very well for clusters of different sizes and densities, as is commonly the case for biological SMLM data. However, in SMLM data, DBSCAN improved the analysis when applied to data from previously defined clusters. In this case, the use of a second round of analysis amended a poor cluster detection, or detected intra-cluster inhomogeneities that arise from subdomains in the distribution of clustered molecules.

As already reported^(^^19^^)^, Voronoi tessellation (Diinamic-V) was robust to multiple detections and different background densities, but for this, the polygon size threshold has to be correctly determined. The existing software relies on the calculation of the mean density outside clusters (SR-Tesseler^(^^18^^)^) or on the simulation of random distributions (ClusterViSu^(^^19^^)^) to set this value. We preferred to introduce a user-defined threshold that considers the labeling method, which heavily affects the expected density of detections. This threshold was then calculated considering the size of the labeled molecule, the expected number of fluorophores and detections per molecule, and the maximum possible distance between the fluorophore and the labeled molecule. An important difference with respect to previously proposed algorithms, Diinamic-V introduced a second density-based selection, using a grid defined by the expected resolution of the data. Our analysis thus pondered the result of Voronoi tessellation with the localization precision. A drawback could be the increase in computational time, therefore a piece of advice could be to systematically compare both on a small subset of data. Nevertheless, Diinamic-V is a good choice when there is an important risk of detection of false clusters due to multiple blinking, and also when the intensity threshold for Diinamic-R is difficult to find, that is, when the density of non-clustered detections is not homogeneous.

One criticism that can be made is the use of multiple user-defined thresholds. Most of the analytical algorithms proposed in the literature need the setting of user-defined thresholds and parameters that can be annoyingly variable from one laboratory to the other or even from one user to the other. Therefore, some strategies were developed, such as the use of Bayesian analysis, to avoid user-defined values^(^^33^^)^. However, biological data are by nature noisy, heterogeneous, and variable, making this blind strategy not easy to apply to all kinds of datasets. Moreover, as reported by Nieves *et al.*^(^^23^^)^, algorithms that rely on more thresholds were more performant. Consequently, we decided to keep the possibility to use thresholds that must be determined for each type of sample that is imaged to assure biological plausibility.

### 5.2. Proposed pipeline to analyze clustering on SMLM data

Even if SMLM techniques may induce various artifacts in clustering analysis, it is possible to minimize these artifacts by carefully preparing the experiment and by managing data correctly. An important point is to decide whether to use parameters to exclude candidate clusters and which parameters to use. The first issue to consider is whether the molecule under study is already known to form clusters. If clusters were already described (i.e., receptors in synapses) then the parameters could be set considering the size of these clusters. Otherwise, the advice would be to keep all the candidate clusters in a first approximation and to compare the results obtained with different labeling strategies to evaluate the artifacts due to the technique itself. The points to consider would be:

- Which labeling method can be used? Which is the expected proportion of molecules that could be detected? Which is the probability of observing multiple detections of the same fluorophore? How do you define a cluster?
- Can the distribution of the molecule be predicted?
  - If no: choose parameters that do not exclude any cluster (setting minimum thresholds to zero and maximum to the largest possible).
  - If yes: calculate the expected density of detections in and out of clusters, given the size of the molecule, whether it is composed or not of labeled subunits, and the expected number of observable fluorophores. Set exclusion parameters given the expected size of clusters.
- Whenever possible, use different labeling strategies and fluorophores to image the same molecule. Compare the results to evaluate artifacts and improve the choice of parameters.

Regarding the choice of analysis strategy, we found that:

- If the density of detections was low, the best results were obtained by relying mainly on the pre-selection by intensity segmentation (Diinamic-R).
- If the density of non-clustered detections was variable (i.e., multiple blinking or non-homogeneous distribution of non-clustered molecules), Diinamic-V was a better choice.
- If the ratio of detections densities in and out clusters was low, the detection of subdomains within clusters could improve the results.

In conclusion, by combining multiple and progressive analysis steps, Diinamic algorithms offer the flexibility needed to adapt the analysis protocol to a large variety of molecular distributions and SMLM data. In addition, they provide the possibility to introduce biology-based criteria (the expected characteristics of clusters) to describe the clustering behavior of molecules.

## Data Availability

All data reported in this paper will be shared upon request. Any additional information required to re-analyze the data reported in this paper is available from the lead contact upon request. A package containing all the tools implemented with Graphic User Interfaces (GUIs), a detailed user manual, and a tool helping the optimization of parameters for executing Diinamic-R and Diinamic-V is freely available at https://github.com/mlrennerfr/Diinamic.

## Supplementary material

For supplementary material accompanying this paper visit https://doi.org/10.1017/S2633903X23000156. click here to view supplementary material
