# Supplementary material for: Introducing Diinamic, a flexible and robust method for clustering analysis in single-molecule localization microscopy
Source: Biol Imaging. 2023 Jul 10;3:e14. doi: 10.1017/S2633903X23000156 (PMC10936397; doi:10.1017/S2633903X23000156)
Supplement: Supplementary file 1 [file S2633903X23000156sup001.pdf]

Supplementary information

Introducing Diinamic, a flexible and robust method for clustering analysis in single-molecule localization microscopy

Anne-Lise Paupiah, Xavier Marques, Zaha Merlaud, Marion Russeau, Sabine Lévi, Marianne Renner.

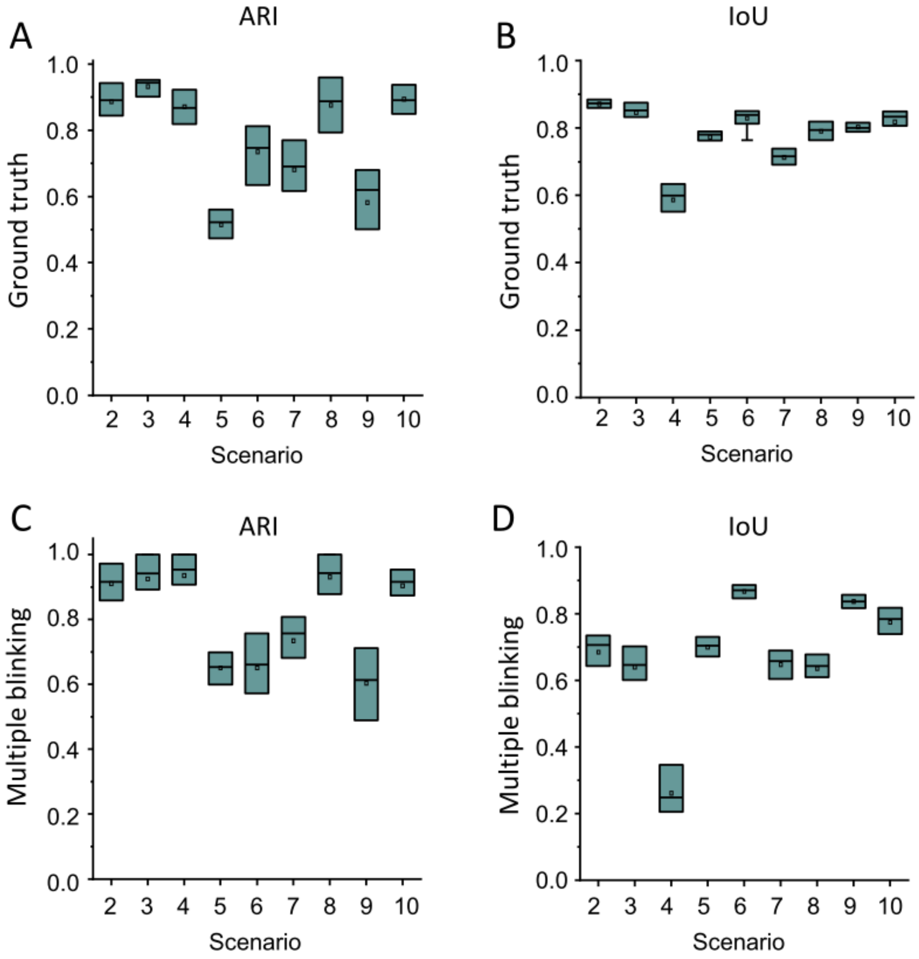

Supplementary Figure S1: Performance analysis of Diinamic-R against Scenarios 2-10 from Nieves and coll. (2023)[23] in absence (Ground truth, A and B) or presence (Multiple blinking, C and D) of multiple detections per original simulated detection. Results were scored using ARI (A,C) and IoU (B,D). Parameters were optimized for the first simulation and then applied for all the simulations (n = 50) from each Scenario. Median and IQR range (25-75%). Open black squares indicate the mean score.

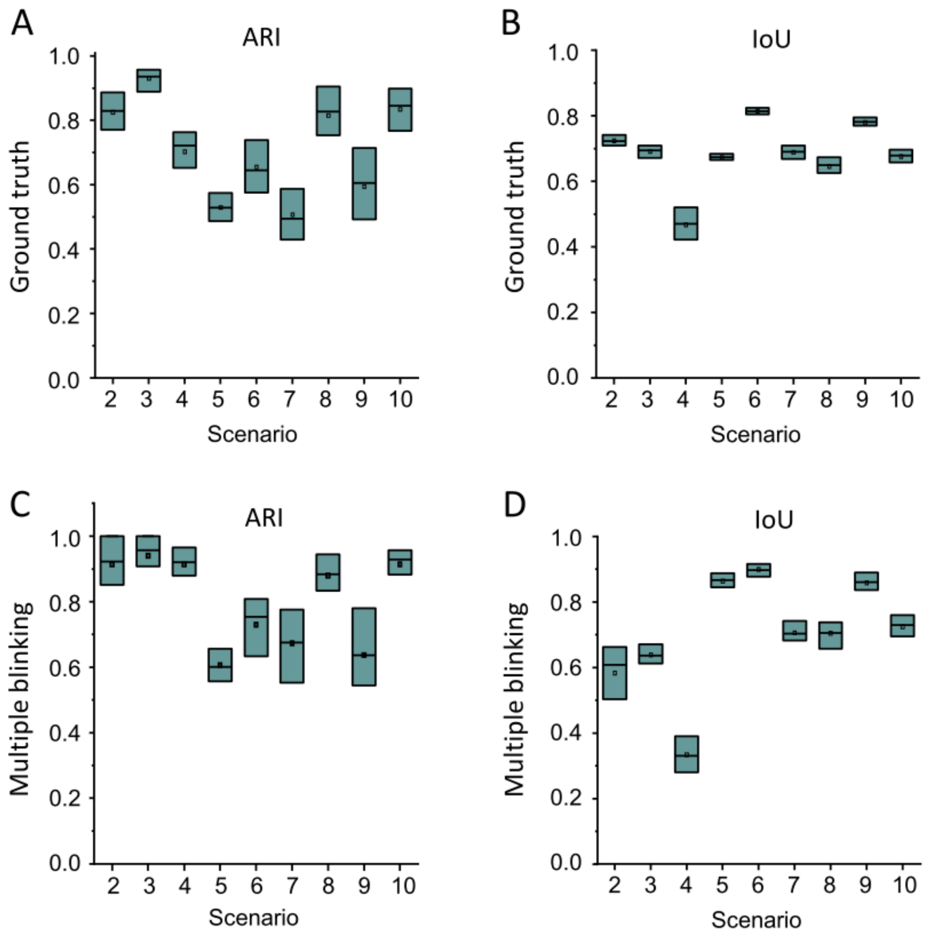

Supplementary Figure S2: Performance analysis of Diinamic-V against Scenarios 2-10 from Nieves and coll. (2023)[23] in absence (Ground truth, A and B) or presence (Multiple blinking, C and D) of multiple detections per original simulated detection. Results were scored using ARI (A,C) and IoU (B,D). Parameters were optimized for the first simulation and then applied for all the simulations (n = 50) from each Scenario. Median and IQR range (25-75%). Open black squares indicate the mean score.

Algorithm	ARI – Ground truth	
	> ~0.8	~0.6-0.8
**Diinamic-R**	5	2
**Diinamic-V**	4	3
**DBSCAN**	4	3
**ToMATo**	4	2
**KDE**	3	2
**CAML**	2	3
**FOCAL**	1	3
**SRTesseler**	1	3
**ClusterVISU**	1	3

Algorithm	ARI – Multiple blinking	
	> ~0.8	~0.6-0.8
**Diinamic-R**	5	3
**Diinamic-V**	5	4
**DBSCAN**	3	2
**ToMATo**	0	5
**KDE**	0	4
**CAML**	0	5
**FOCAL**	0	1
**SRTesseler**	0	3
**ClusterVISU**	0	4

Algorithm	IoU – Ground truth	
	> ~0.8	~0.6-0.8
**Diinamic-R**	2	6
**Diinamic-V**	0	8
**DBSCAN**	1	5
**ToMATo**	0	6
**KDE**	0	4
**CAML**	0	6
**FOCAL**	0	4
**SRTesseler**	0	1
**ClusterVISU**	0	0

Algorithm	IoU – Multiple blinking	
	> ~0.8	~0.6-0.8
**Diinamic-R**	2	6
**Diinamic-V**	0	7
**DBSCAN**	0	7
**ToMATo**	0	5
**KDE**	0	3
**CAML**	0	0
**FOCAL**	0	1
**SRTesseler**	0	2
**ClusterVISU**	0	0

Supplementary Tables S1: Number of scenarios with scores over ~ 0.8 or between ~0.6 and ~0.8 for ARI and IoU scores, for the indicated algorithms and datasets (score values from Supplementary Fig. S1 and 2 and from Nieves et al, 2023[23]).
